# The Anti-Cancer Multikinase Inhibitor Sorafenib Impairs Cardiac Contractility by Reducing Phospholamban Phosphorylation and Sarcoplasmic Calcium Transients

**DOI:** 10.1038/s41598-018-23630-w

**Published:** 2018-03-28

**Authors:** Christopher Schneider, Markus Wallner, Ewald Kolesnik, Viktoria Herbst, Heinrich Mächler, Martin Pichler, Dirk von Lewinski, Simon Sedej, Peter P. Rainer

**Affiliations:** 1 Division of Cardiology, Medical University of Graz, Graz, Austria; 2 Cardiovascular Research Center, Lewis Katz School of Medicine, Temple University, Philadelphia, PA USA; 3 Division of Cardiac Surgery, Medical University of Graz, Graz, Austria; 4 Division of Oncology, Medical University of Graz, Graz, Austria; 5 Department of Experimental Therapeutics, UT MD Anderson Cancer Center, Houston, TX USA; 6 BioTechMed Graz, Graz, Austria; 7 Division of Cardiology, Johns Hopkins University School of Medicine, Baltimore, MD USA

## Abstract

Tyrosine-kinase inhibitors (TKIs) have revolutionized cancer therapy in recent years. Although more targeted than conventional chemotherapy, TKIs exhibit substantial cardiotoxicity, often manifesting as hypertension or heart failure. Here, we assessed myocyte intrinsic cardiotoxic effects of the TKI sorafenib and investigated underlying alterations of myocyte calcium homeostasis. We found that sorafenib reversibly decreased developed force in auxotonically contracting human myocardia (3 µM: −25 ± 4%, 10 µM: −29 ± 7%, 30 µM: −43 ± 12%, p < 0.01), reduced peak cytosolic calcium concentrations in isolated cardiomyocytes (10 µM: 52 ± 8.1% of baseline, p < 0.001), and slowed cytosolic calcium removal kinetics (RT50, RT10, Tau, p < 0.05). Beta-adrenergic stimulation induced augmentation of calcium transient (CaT) amplitude was attenuated in sorafenib-treated cells (2.7 ± 0.3-fold vs. 3.6 ± 0.2-fold in controls, p < 0.001). Sarcoplasmic reticulum (SR) calcium content was reduced to 67 ± 4% (p < 0.01), and SR calcium re-uptake slowed (p < 0.05). Sorafenib significantly reduced serine 16 phosphorylation of phospholamban (PLN, p < 0.05), while PLN threonine 17 and CaMKII (T286) phosphorylation were not altered. Our data demonstrate that sorafenib acutely impairs cardiac contractility by reducing S16 PLN phosphorylation, leading to reduced SR calcium content, CaT amplitude, and slowed cytosolic calcium removal. These results indicate myocyte intrinsic cardiotoxicity irrespective of effects on the vasculature and chronic cardiac remodeling.

## Introduction

Targeted therapies, such as monoclonal antibodies, small molecule protein kinase inhibitors, or immunotherapy revolutionized cancer treatment and substantially improved survival in many types of cancer. Due to the expression of target molecules in non-cancer tissues, however, mechanism-based therapies exhibit substantial side effects. Cardiotoxicity is a major concern with these drugs, particularly if pre-existing cardiac conditions are present^1^. In contrast to conventional chemotherapies that have been studied over decades, however, knowledge on the toxicity of these emerging compounds is still limited. The number of drugs available is growing rapidly, as are the number of patients and associated adverse events. This highlights the importance of studying novel cancer-drug related cardiotoxicity and was also evidenced by the recent development of cardio-oncology guidelines by major scientific societies^2^.

Tyrosine kinase inhibitors (TKIs) are small molecules that block kinases involved in tumor growth and angiogenesis. Imatinib, which inhibits ABL1 kinase, was the first such agent and transformed the treatment of chronic myeloid leukemia (CML)^3,4^. Currently, more than 20 small molecule kinase inhibitors are FDA approved^5^. Many kinase inhibitors bind to the ATP pocket region of the active and/or inactive kinase^6^. As this region is similar in many kinases, one agent may inhibit multiple kinases. Examples are the multi-kinase inhibitors sunitinib and sorafenib that inhibit more than 50 and 15 kinases, respectively^7^. Such multi-kinase targeting enhances anti-proliferative properties but may also increase the risk of side effects. In addition, kinase inhibitors may impact other signaling molecules such as enzymes^8^, or cardiac mitochondrial function^9,10^, which may also contribute to cardiotoxic effects.

Multi-kinase inhibitors blocking vascular endothelial growth factor (VEGF) signal transduction appear to be particularly problematic in respect to cardiovascular toxicity^11^. They often increase blood pressure (22% with sunitinib^12^, 15% with sorafenib)^13^, and increase the risk of vascular events such as endothelial injury, vasospasm, or arterial thrombosis^14,15^. If pre-existing cardiac conditions like hypertrophy or ischemia are present, the adverse effects of multi-kinase inhibition with sorafenib are amplified^16^. In addition to negative effects on vascular function and events, several studies demonstrated impairment of myocardial function and development of heart failure in a significant number of patients^10,17,18^. Interestingly, the remodeling heart shares many signaling pathways with cancer as it undergoes hypertrophy, angiogenesis, and cell death^19^, which may explain the high incidence of cardiotoxicity.

In the current study, we investigated the multi-kinase inhibitor sorafenib (trade name Nexavar®). Sorafenib is a tyrosine kinase inhibitor with anti-VEGF activity and was first approved by the FDA in 2005. Among other kinases it inhibits VEGF receptor 1–3 tyrosine kinases, platelet derived growth factor (PDGF) family receptors, and the Ras-Raf-MEK-ERK signal transduction pathway^1,6^. Sorafenib may also inhibit serine-threonine kinases like the cyclin-dependent kinase family^6^. Currently, sorafenib is used to treat advanced renal cell carcinoma (RCC), unresectable hepatocellular carcinoma (HCC), and advanced differentiated thyroid carcinoma (DTC). Typically, sorafenib is continuously used to treat these tumors and as with other TKIs, ample cardiotoxic effects have been described: sorafenib substantially increased arterial blood pressure, the incidence of cardiac ischemia/infarction, and congestive heart failure^2,20–22^. However, it remains unclear if cardiac toxicity is mainly a consequence of vascular effects with secondary myocardial damage, impaired remodeling with chronic treatment, or direct cardiomyocyte toxic effects.

Here, we aimed to investigate sorafenib’s acute effects on myocardial contractility in the absence of vascular influences or chronic treatment-induced remodeling. We found acute, profound, and reversible negative inotropy in human tissue and describe the underlying changes in cardiomyocyte intrinsic calcium handling.

## Results

### Acute negative inotropy in human myocardium

**Figure 1:**
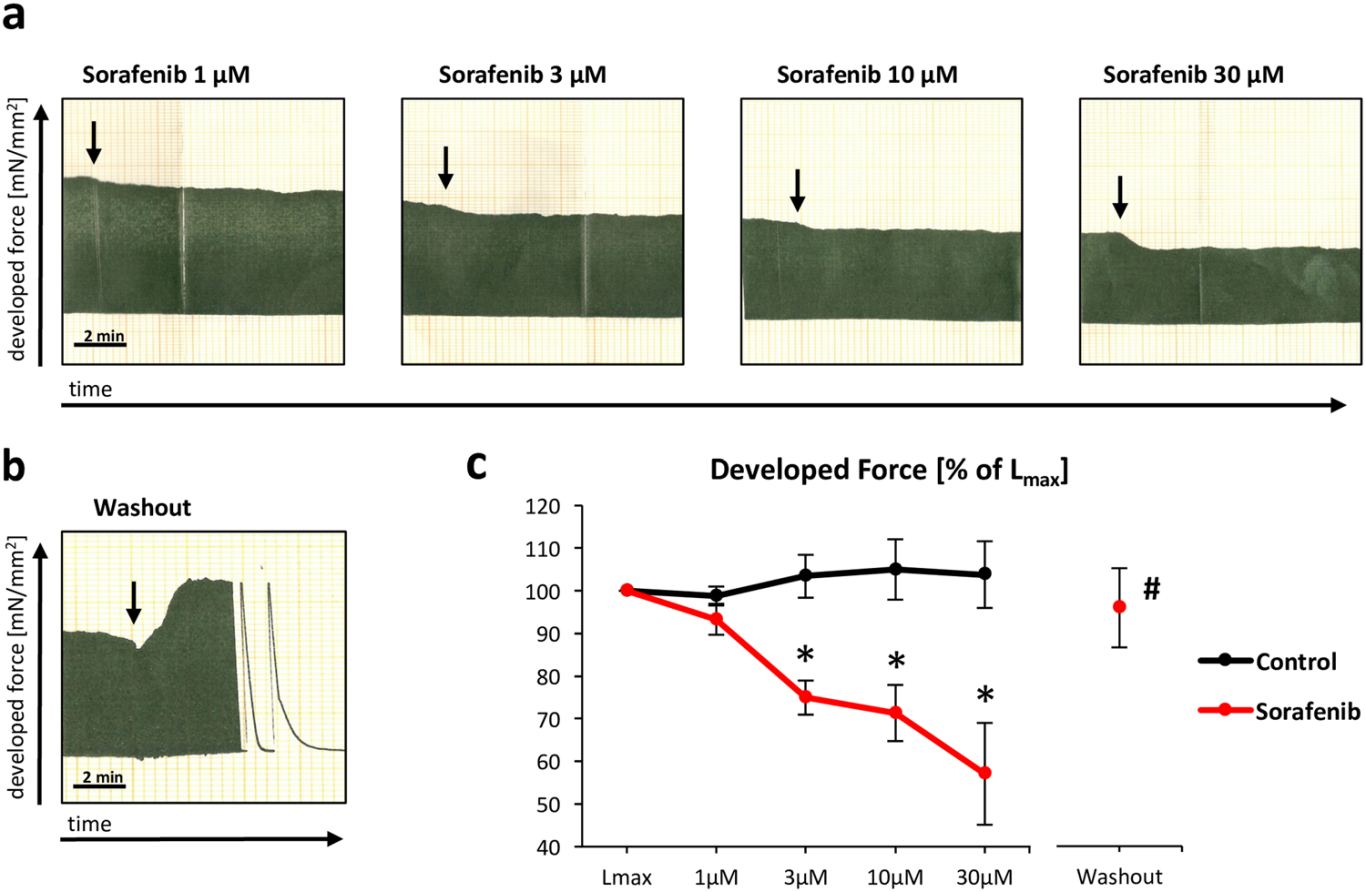
Concentration-dependent decline of cardiac contractility in auxotonically contracting human atrial trabeculae upon sorafenib administration. Original analogue recordings of developed force with increasing (1–30 µM) concentrations of sorafenib (**a**) and after sorafenib wash-out (**b**). Summary data (**c**). n = 9/10 trabeculae (control/sorafenib) from 11 hearts; *p < 0.05 vs. baseline (L_max_) by Friedman’s repeated measures one-way ANOVA on ranks (ANOVA p < 0.001) and p < 0.01 vs. control by Student’s t-test, ^#^p < 0.05 vs. 30 µM sorafenib and p = 0.68 vs. baseline (L_max_) by paired t-test.

Sorafenib rapidly reduced developed systolic force in human right atrial appendage trabeculae in a concentration-dependent manner (3 µM: −25 ± 4%, 10 µM: −29 ± 7%, 30 µM: −43 ± 12%, all p < 0.01 vs. control; Fig. 1). The negative effect on myocardial contractility was reversed by removing sorafenib from the perfusate (p = 0.01 vs. 30 µM, p = 0.68 vs. L_max_) and sustained over several hours (Supplementary Fig. 1). Control treatment with vehicle alone (DMSO) did not reduce developed systolic force.

### Depressed cytosolic calcium transients and attenuated beta-adrenergic reserve

**Figure 2:**
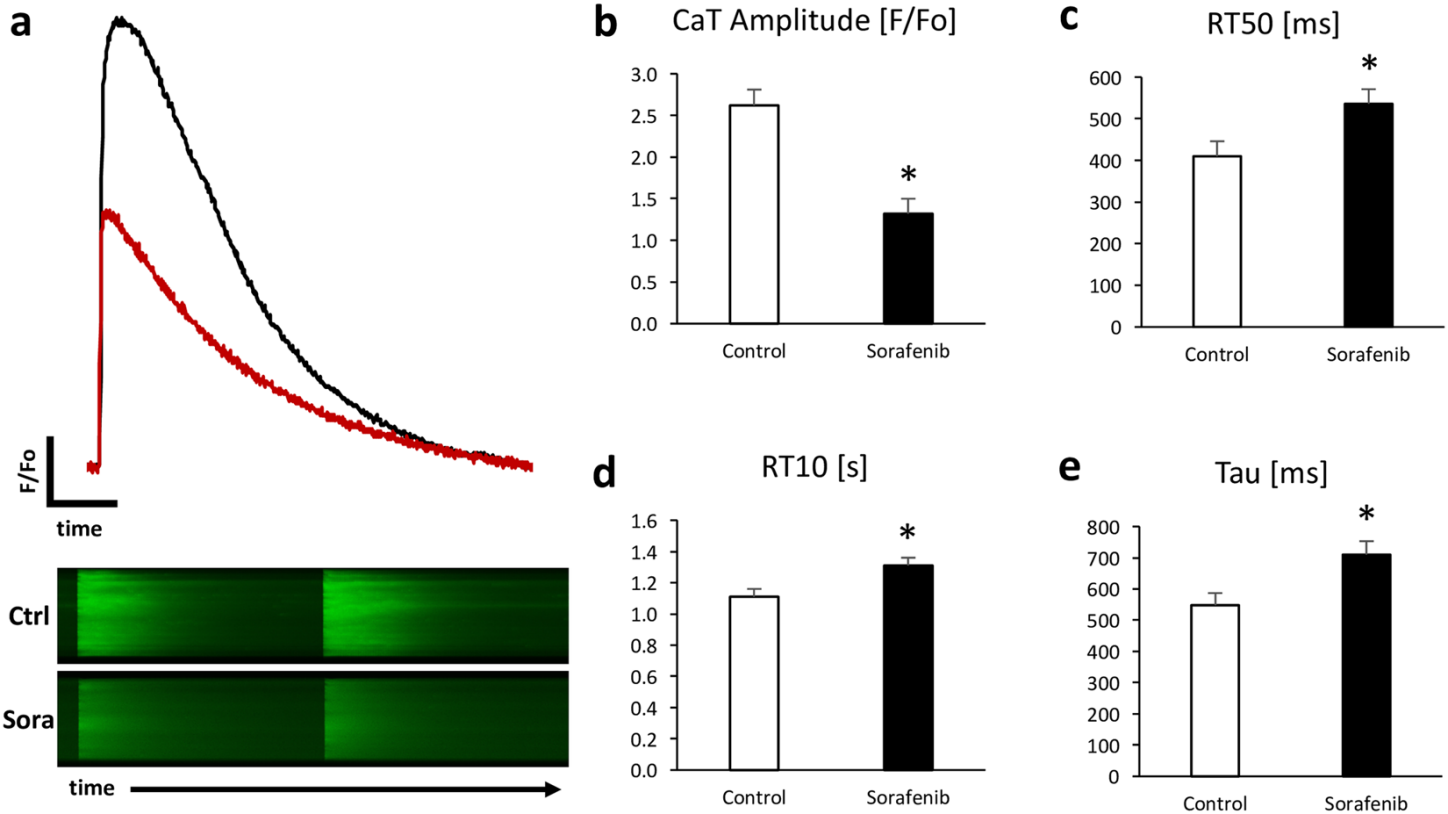
Reduced calcium transient amplitude and slowed calcium re-uptake in sorafenib (10 µM) treated murine ventricular cardiomyocytes. Representative calcium transients (above) and original 2D calcium wave line-scan (below) (**a**) and summary data for calcium transient amplitude (**b**), time to 50 and 90% (RT10) decay of calcium transient signal (**c,d**), and relaxation constant Tau (**e**). n = 17 cells/10 hearts, *p < 0.001 for treatment/time interaction and p < 0.05 vs. control by 2-way mixed ANOVA with Welch’s post hoc t-test.

We next assessed sorafenib’s effects on calcium homeostasis and beta-adrenergic responsiveness in isolated murine ventricular cardiomyocytes. Sorafenib significantly reduced peak systolic calcium amplitude to 50 ± 6.9% of controls (p < 0.001, n = 17 cells/10 hearts, 95% CI 37–64%, Fig. 2a,b). Sorafenib administration also slowed cytoplasmic calcium removal as determined by a significant prolongation of the time to 50% and 90% decline of calcium transients, and prolongation of the time constant of calcium decay Tau (Fig. 2c–e).

**Figure 3:**
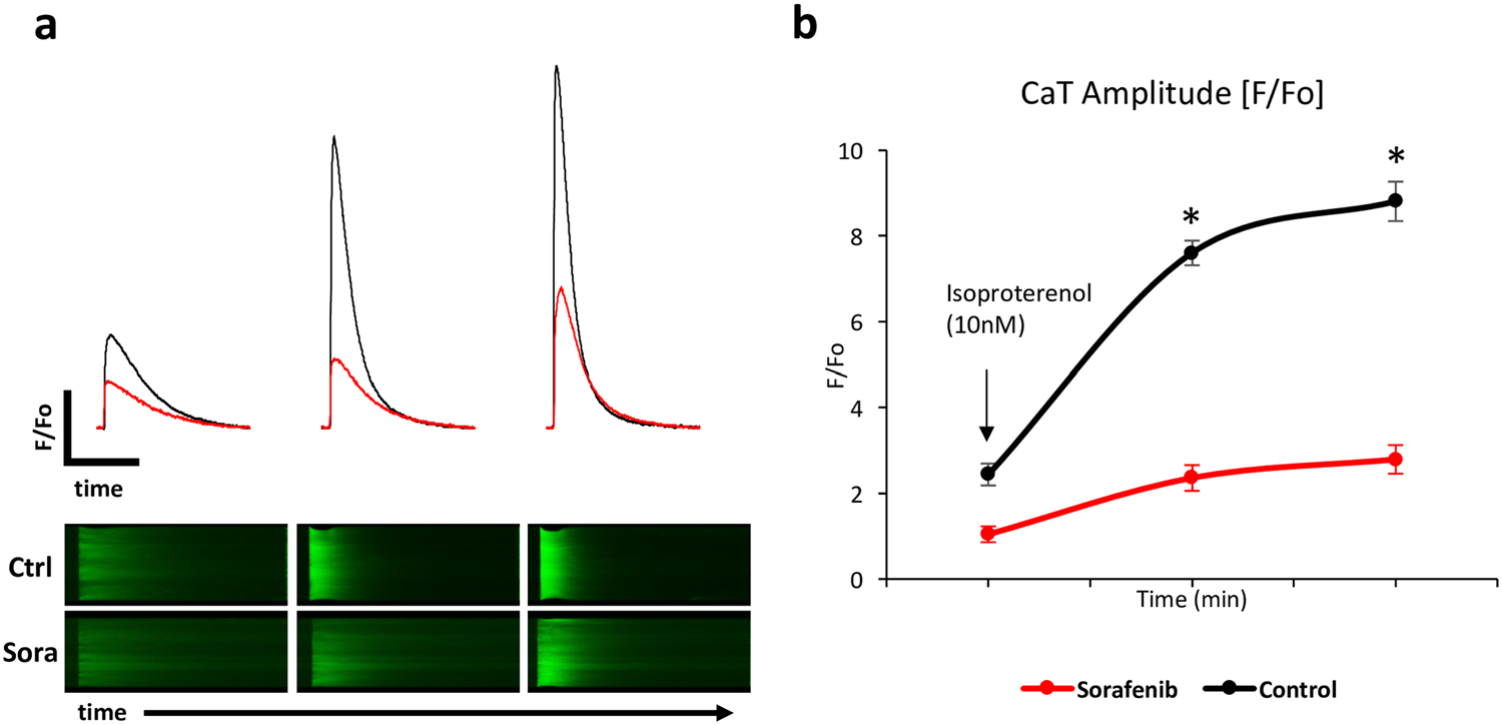
Attenuated beta-adrenergic responsiveness in sorafenib (10 µM) treated cardiomyocytes exposed to isoproterenol. Representative calcium transients (above), original 2D calcium wave line-scan (below) (**a**), and summary data (**b**). n = 14–15 cells/3–5 hearts per time point. *p < 0.001 for treatment/time interaction and p < 0.005 vs. control by 2-way mixed ANOVA with Welch’s post hoc t-test.

Next, we tested beta adrenergic reserve using isoproterenol, a beta-1 and 2 adrenergic agonist, in myocytes exposed to sorafenib. In control cells, isoproterenol induced a 3.6 ± 0.2-fold (95% CI 3.2–4.0) increase in CaT amplitude, while this response was attenuated to 2.7 ± 0.3-fold (95% CI 2.0–3.3) in sorafenib treated myocytes (p < 0.001 for interaction, n = 14–15 cells/3–5 hearts; Fig. 3). Thus, sorafenib reduced the isoproterenol-induced increase in peak cytoplasmic systolic calcium concentrations.

### Reduced SR calcium load and slowed SR calcium re-uptake

**Figure 4:**
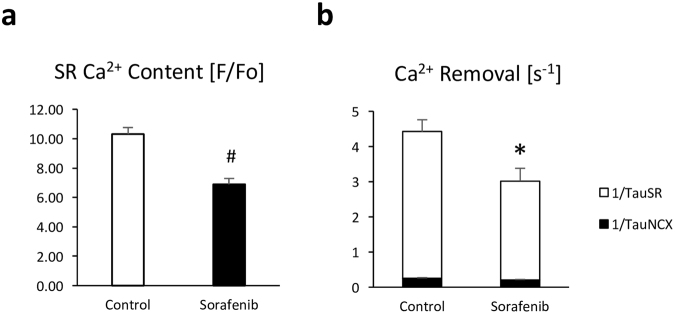
Reduced SR calcium content and cytosolic calcium removal in sorafenib treated cardiomyocytes. Sorafenib (10 µM) reduces SR calcium content as assessed by rapid caffeine (30 mM) application (**a**), and reduces the rate constant of cytosolic calcium removal through SERCA (1/Tau_SR_) (**b**). n = 3–10 cells/3–5 hearts. *p < 0.05 and #p < 0.01 vs. control by t-test.

During the cardiac cycle, calcium is released into the cytosol from the sarcoplasmic reticulum (SR) in large quantities to increase cytosolic calcium and initiate contraction. Thus, we next assessed SR calcium content by inducing near total calcium release from the SR with caffeine. The ensuing caffeine-induced calcium transient (CaT_caff_) is an estimate of SR calcium content. CaT_caff_ was substantially reduced in sorafenib pre-treated cardiomyocytes to 67 ± 4% (95% CI 59–74%) of control cells (p < 0.01, n = 10 cells/3–5 hearts; Fig. 4a) indicating reduced SR calcium load. The rate constant of cytoplasmic calcium removal through the SR calcium ATPase (SERCA, 1/Tau_SR_) was significantly reduced in these cells (2.81 ± 0.36 s^−1^ vs. 4.16 ± 0.34 s^−1^ in controls, p < 0.05, 95% CIs 2.15–3.47 and 3.45–4.87; Fig. 4b), while calcium removal by the Na^+^/Ca^2+^ exchanger (NCX, 1/Tau_NCX_) was not significantly altered.

### Decreased phospholamban phosphorylation at serine 16

**Figure 5:**
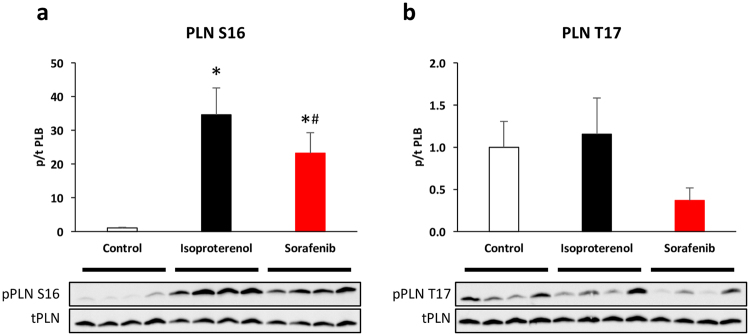
Decreased phosphorylation of phospholamban (PLN) at serine 16 in sorafenib (10 µM) treated cardiomyocytes. Isoproterenol (10 nM) induced phosphorylation of phospholamban at serine 16 is attenuated by sorafenib (**a**). Threonine 17 phosphorylation is borderline significantly (p = 0.098) reduced (**b**). Summary data (above) and representative blots (below). n = 6 hearts per group. *p < 0.05 vs. control and #p < 0.05 vs. isoproterenol by Kruskal Wallis ANOVA (ANOVA p = 0.013). For complete western blots see Supplementary Fig. S4.

The activity of SR calcium re-uptake through SERCA2a is regulated by its interaction with the regulatory protein phospholamban (PLN). Phosphorylation of phospholamban by protein kinase A (PKA) at serine 16 and/or calcium/calmodulin-dependent kinase II (CamKII) at threonine 17 relieves phospholamban-SERCA2a inhibition and increases SERCA2a calcium affinity^23^. We found significantly reduced phosphorylation of phospholamban at the serine 16 phosphosite (p < 0.05, n = 6 per group, Fig. 5a), while decreased CaMKII-dependent phosphorylation at threonine 17 did not reach statistical significance (p = 0.098, n = 6 per group; Fig. 5b). CaMKII itself was not altered in total or activity-indicating phospho-T286 expression (p = 0.34, n = 5 per group; Supplementary Fig. S2). Sorafenib also reduced PLB S16 phosphorylation without isoproterenol stimulation (Supplementary Fig. S3). The expression of other key calcium handling proteins (ryanodine receptor (RyR), NCX, SERCA2a) was not altered. Further, sorafenib did not significantly impact the phosphorylation of the PKA targets RyR S2808 and troponin I (TnI) S23/24. Likewise, phosphorylation of the serine-threonine protein phosphatase 1 alpha (PP1α) at phospho-threonine 320, which can be phosphorylated by cyclin-dependent kinase 2, was not changed (Supplementary Fig. S3).

## Discussion

Here, we show that sorafenib exerts rapid, concentration-dependent, and reversible negative inotropy in human myocardia. These effects on contractility are cardiomyocyte intrinsic and mediated by a reduction of systolic cytoplasmic calcium concentrations associated with reduced SR calcium load and phospholamban S16 phosphorylation.

Therapy limiting cardiotoxicity is one of the main concerns in cancer treatment with tyrosine kinase inhibitors. Sorafenib causes several cardiac adverse events including hypertension, vascular events leading to ischemia, or congestive heart failure^2,20–22^. Some of these adverse events are likely reversible, particularly if functional impairment occurs without cell death. For example, reduced left ventricular (LV) systolic function and congestive heart failure might improve after treatment stop. This has been shown for another targeted anti-cancer therapy, anti-Her2/neu inhibition with trastuzumab^24^ and contrasts classic cardiotoxicity such as heart failure that manifests dose-dependently upon anthracycline treatment^25^. However, TKIs are often used for continuous treatment of otherwise therapy resistant cancers. Hence, there is a lack of evidence on the reversibility and prognosis of manifest cardiotoxicity with long-term treatment. Even if reversible, cardiotoxicity poses major challenges when evaluating the risk of cardiotoxicity versus treatment benefit. In addition, some cardiac adverse events such as vascular events leading to ischemia are often not reversible. In the case of acute myocardial infarction, sorafenib appeared to be particularly damaging to post-infarction healing in preclinical models^16^.

Adverse events are typically evaluated during chronic treatment. Thus, it is often impossible to distinguish the contribution of different cell types and underlying mechanisms. For example, it remains hard to distinguish intrinsic myocardial side effects from secondary myocardial damage caused by vascular events such as hypertension, endothelial dysfunction, vasospasm, or thromboembolism. The adverse effects on myocardial function are likely particularly relevant in multikinase inhibitors with anti-VEGFR activity and may be due to limited angiogenesis and/or direct protective effects of VEGF signaling in chronic cardiac remodeling^26,27^. However, there remains some controversy on direct intrinsic myocardial toxicity of these agents. Duran *et al*.^16^ for example found that sorafenib did not impact ejection fraction in a murine myocardial infarction model or fractional shortening of isolated feline cardiomyocytes. They found that sorafenib rather changed the rate of apoptosis and proliferation of c-kit+ stem cells in the infarcted heart. In the present study, we show that sorafenib does acutely impact human myocardial contractility and isolated cardiomyocyte calcium handling. This is consistent with our previous work^28^, in which we found comparable effects for the related TKI sunitinib, and suggests a class effect for multikinase inhibitors. Recently, Sharma and colleagues devised high-throughput screening of human-induced pluripotent cardiac stem cells (hiPSC)^29^. HiPSC derived cardiomyocytes were assessed in terms of viability, contraction, calcium handling, and electrophysiology. Sorafenib and vemurafenib were the most cardiotoxic drugs of 22 TKIs investigated in this study and reduced contractility and viability at a concentration of sorafenib that was similar or lower to the concentration that we used in our experiments. Together, these data clearly support cardiomyocyte intrinsic toxicity irrespective of chronic effects on the vasculature or myocardial remodeling.

Sorafenib’s role in acutely blunting beta-adrenergic induced contractile reserve is interesting. While this effect may acutely reduce contractility, the long-term consequences are less clear. In heart failure, cardiac specific beta-1 blockade is protective and there is some evidence that cardiac selective beta blockers are useful in reducing cardiotoxic events in breast cancer patients treated with anthracyclines and the anti-HER2/neu monoclonal antibody trastuzumab^30,31^. Intriguingly, beta blockade also appears to exhibit anti-tumor effects in some cases thereby enhancing the efficacy of cancer treatment in retrospective analyses^32^. Although prospective studies are warranted to support this notion, these observations strengthen the rationale of cardioprotective co-treatment. Another layer of complexity is added by differential consequences of cardiac beta-1 vs. beta-2 signaling that also appear to be important in trastuzumab’s cardiac effects^33^, and the fact that sorafenib rapidly reduced phosphorylation of PLB S16, while the phosphorylation of the other PKA targets TnI S23/24 and RyR S2808 was not altered at this early time point.

### Limitations

As with all pathophysiologic signaling, the combined responses of different cell types and tissues over chronic treatment periods in patients with individual pre-existing cardiac and extra cardiac conditions determines the net effect of sorafenib’s cardiac effects and thus the clinical outcome. We determined acute and myocyte-intrinsic reversible effects of sorafenib on cardiac contractility. In addition to these effects, chronic treatment with sorafenib decreases cell survival and proliferation and thus may propagate irreversible cardiac remodeling. Further, TKIs are multikinase inhibitors and it is probable that other kinases and signaling pathways that we have not investigated here contribute to their long-term effects on the heart. In an unbiased kinome scan of 442 target proteins e.g., sorafenib reduced the activity of 54 kinases to an activity of less than 5% compared to DMSO controls^34,35^. Understanding discrete aspects of the underlying pathophysiology in reductionist models, such as the direct cardiomyocyte effects of TKIs, may help in early recognition of critical adverse events, identification of potential protective co-treatments, and ideally aid the design of future agents with less cardiotoxic potential.

In conclusion, we report acute negative inotropy of the tyrosine kinase inhibitor sorafenib in human myocardia that is associated with decreased cytosolic calcium concentrations, SR calcium load, and reduced phospholamban S16 phosphorylation, evidencing direct cardiomyocyte-intrinsic toxicity of sorafenib.

## Methods

### Human myocardium

Functional experiments were performed in right atrial appendage tissue that was routinely excised to enable cannulation for extracorporeal circulation (n = 15 hearts). Five patients were females, 9 patients required bypass surgery, 2 aortic valve replacement, 3 combined aortic valve and bypass surgery, and one patient combined mitral valve and bypass surgery. Average left ventricular ejection fraction was 55 ± 7%. The mean age of patients was 66 ± 4 years. Average body-mass-index (BMI) was 28 ± 2 kg/m^2^. Medication included statins in all patients, ACE-inhibitors in 7 patients, angiotensin II receptor blockers (ARBs) in 4 patients, and beta blockers in 11 patients.

The study was approved by the ethics committee of the Medical University Graz, conformed to all relevant guidelines, regulations, and the declaration of Helsinki, and all patients gave written informed consent to participate in the study.

### Muscle strip preparation

Appendages were transported to the lab in ice cold cardioplegic solution containing (in mM): Na^+^ 152, K^+^ 3.6, Cl^−^ 135, HCO_3_^−^ 25, Mg^2+^ 0.6, H_2_PO_4_^−^ 1.3, SO_4_^2−^ 0.6, Ca^2+^ 0.2, glucose 11.2 and 2,3-butanedione-monoxime (BDM) 30, equilibrated with 95% O_2_ and 5% CO_2_ to a pH of 7.4. This solution has been shown to protect the myocardium during transportation and from cutting injury at the time of dissection with full reversibility of the cardioplegic effects upon washout. Small endocardial trabeculae (cross-sectional area <0.5 mm^2^) were dissected with the help of a stereo-microscope. Muscle strips were then mounted in organ chambers between miniature hooks, connected to an isometric force transducer (Scientific Instruments, Germany), and superfused with modified Tyrode’s solution (37 °C) of the composition given above except that BDM was omitted and [Ca^2+^]_o_ stepwise increased to 2.5 mM. Muscle strips were electrically field stimulated at 1 Hz, and isometric contractions were recorded at optimum preload (L_max_).

### Drugs

Sorafenib (BAY 43-9006) was purchased from BioVision (Milpitas, CA), dissolved in DMSO, and freeze-stored in stock aliquots. The stock solution was added to the perfusate to achieve a final concentration of 1–30 µM with a constant concentration of 0.1% DMSO. Isoproterenol and caffeine were administered at a final concentration of 10 nM and 30 mM, respectively. If not otherwise stated, all chemicals were obtained from Sigma-Aldrich (Vienna, Austria).

### Murine cardiomyocyte isolation

Ventricular cardiomyocytes were freshly isolated from adult male C57BL/6 J mice (The Jackson Laboratory, Bar Harbor, ME, USA and Charles River, Sulzfeld, Germany) at 12–25 weeks of age. All animal procedures were performed in strict accordance with requirements pertaining work with experimental animals on the European Union, national, and institutional levels and approved by the institutional animal care and use committee (IACUC) at the Medical University of Graz, Austria (Division for Biomedical Research, Organizational Unit for Research Infrastructure). Mice were housed in a dedicated facility with 12 h light/dark regime and unrestricted access to food and water under the care of certified technicians and veterinarians. Mice were anesthetized using isoflurane and heparinized (50 IU) prior to cervical dislocation and organ removal. The heart was removed, rinsed in isolation solution containing (in mM): NaCl 135, KCl 4.7, KH_2_PO_4_ 0.6, Na_2_HPO_4_ 0.6, MgSO_4_ 1.2, HEPES 10, taurine 30, 2,3-butanedione monoxime 10, glucose 10. pH was adjusted to 7.4 at room temperature with NaOH. Then, the ascending aorta was rapidly cannulated and the heart perfused on a Langendorff setup (Radnoti, Monrovia, CA, USA) at constant flow at 37 °C with perfusion solution and subsequently with enzyme solution of the same composition as above plus the addition of Liberase TM (Roche), Trypsin, and CaCl_2_ 12.5 µM. After digestion, ventricles were removed, transferred in enzyme free isolation solution containing 10% bovine calf serum (BCS) and further dissected. Dissociated cells were filtrated through a 250 µm nylon-mesh. Then, the calcium concentration was stepwise increased to 1 mM in a tyrode solution containing (in mM): NaCl 136, KCl 5, CaCl_2_ 1, MgCl_2_ 1, HEPES 10, glucose 10; pH = 7.40.

### Calcium transient measurements

Cardiac myocytes were then loaded with 1.5 µM of the calcium-sensitive fluorescent dye Fluo-4/AM (Life Technologies, Carlsbad, CA, USA), washed, and seeded on laminin-coated glass bottom dishes. Cells were superfused at constant flow rates using a gravity-driven perfusion system connected to a valve controller (VC-8, Warner Instruments, Hamden, CT, USA) and electrically stimulated at 0.5 Hz (MyoPacer field stimulator, Ionoptix, Westwood, MA, USA). Calcium transients were recorded using a Zeiss laser scanning microscope (LSM 700, Zeiss, Jena, Germany) equipped with a 40  oil-immersion objective lens in line scan mode (Zen Software v5.5.0.375, Zeiss). Fluo-4 was excited using 488 nM laser light (Lasos – Laser Rack LSM 700) and the emitted fluorescence was recorded at >505 nM. Fluo-4 fluorescence was expressed as normalized changes in background-corrected fluorescence emission (F/F_0_) with diastolic fluorescence at the begin of an experiment defined as F_0_. Only rod-shaped cells with clear striations and quiescence without pacing were used for experiments. SR calcium content was assessed by rapid application of 30 mM caffeine after stopping field stimulation. Calcium removal rate constants were calculated according to Tocchetti and colleagues^36^ as 1/Tau_twitch_ = 1/Tau_NCX_ + 1/Tau_SR_.

### Immunoblots

Freshly isolated murine cardiomyocyte suspension was incubated for 5 minutes with DMSO (0.1%) or sorafenib (10 µM), then treated for 10 minutes with isoproterenol (10 nM), centrifuged, and the pellet snap frozen in liquid nitrogen. Protein concentration was estimated using BCA assay (Pierce, Thermo Fisher Scientific, Waltham, MA, USA) according to the manufacturer’s protocol.

Protein lysates were reduced and denatured and 20–30 µg of protein loaded on 16.5% Tris-Tricine (PLB) or 4–12% Bis-Tris (CamKII) gels (Criterion, BioRad, Vienna, Austria). Transfer to nitrocellulose (PLB) or PVDF (CamKII) membranes (Amersham, GE, Vienna, Austria) was performed for 1–2 hours using a Criterion Blotter (BioRad) at 4 °C. Membranes were stained using Ponceau solution to control transfer, washed, and blocked using 5% milk in TBST. Primary antibodies were incubated at 4 °C overnight and secondary antibodies for 50 minutes at 4 °C. PLB, RyR S2808, and SERCA 2a antibodies were obtained from Badrilla (Leeds, UK; catalog numbers A01014, A01012, A01013AP, A01030, A01020). CamKII, TnI, GAPDH, RyR, and PP1α antibodies were obtained from Abcam (Cambridge, UK; ab32678, ab47003, ab2827) and Cell Signaling Technologies (Leiden, The Netherlands; CST4436, CST5174, CST4004, CST2581). The NCX antibody was obtained from Abnova (Taipei, Taiwan; MAB1789). Secondary antibodies were obtained from GE Healthcare. Membranes were developed (Clarity Western ECL or Super Signal West Femto, Thermo Fisher Scientific) and imaged on a ChemiDoc Touch System (BioRad). Band intensities were determined using ImageJ software (NIH) after subtraction of in-lane background and exported to R for statistical analyses.

### Statistical Analyses

Statistical analysis was performed using GraphPad Prism and R Studio (RStudio v0.99.491, packages readxl, plyr, ggplot2, nlme, lme4, XLConnect). Normality was tested using Shapiro-Wilk test and homogeneity of variance using Bartlett’s test. Statistical comparisons were performed using Student’s t-test, one- or two-way analysis of variance (ANOVA), Mann-Whitney U test, Kruskal Wallis ANOVA, or Friedman repeated measurements ANOVA on ranks as appropriate. Post hoc testing was performed using false discovery rate (FDR) adjusted paired t-test (Benjamini Hochberg procedure), Dunnett’s test, or Welch’s t-test. The respective statistical tests are indicated in the figure legends. Data are presented as mean ± SEM. p values refer to comparisons on the cardiomyocyte level unless otherwise given and were considered statistically significant if <0.05.

### Data availability

All data generated or analyzed during this study are included in this published article (and its Supplementary Information files).

## Electronic supplementary material

Supplementary Material
