# Supplementary material for: The Anti-Cancer Multikinase Inhibitor Sorafenib Impairs Cardiac Contractility by Reducing Phospholamban Phosphorylation and Sarcoplasmic Calcium Transients
Source: Sci Rep. 2018 Mar 28;8:5295. doi: 10.1038/s41598-018-23630-w (PMC5871797; doi:10.1038/s41598-018-23630-w)
Supplement: Supplementary file 1 — Supplementary Material [file 41598_2018_23630_MOESM1_ESM.pdf]

Supplementary Information

The Anti-Cancer Multikinase Inhibitor Sorafenib Impairs Cardiac Contractility by Reducing Phospholamban Phosphorylation and Sarcoplasmic Calcium Transients

Christopher Schneider[1], Markus Wallner[1,2], Ewald Kolesnik[1], Viktoria Herbst[1], Heinrich Mächler[3], Martin Pichler[4,5], Dirk von Lewinski[1], Simon Sedej[1,6], Peter P. Rainer[1,7*]

[1] Division of Cardiology, Medical University of Graz, Austria
[2] Cardiovascular Research Center, Lewis Katz School of Medicine, Temple University, Philadelphia, PA, USA
[3] Division of Cardiac Surgery, Medical University of Graz, Austria
[4] Division of Oncology, Medical University of Graz, Austria
[5] Department of Experimental Therapeutics, UT MD Anderson Cancer Center, Houston, TX, USA
[6] BioTechMed Graz, Austria
[7] Division of Cardiology, Johns Hopkins University School of Medicine, Baltimore, MD, USA
* Corresponding author, email: herzforschung@gmail.com

Supplementary Figure S1

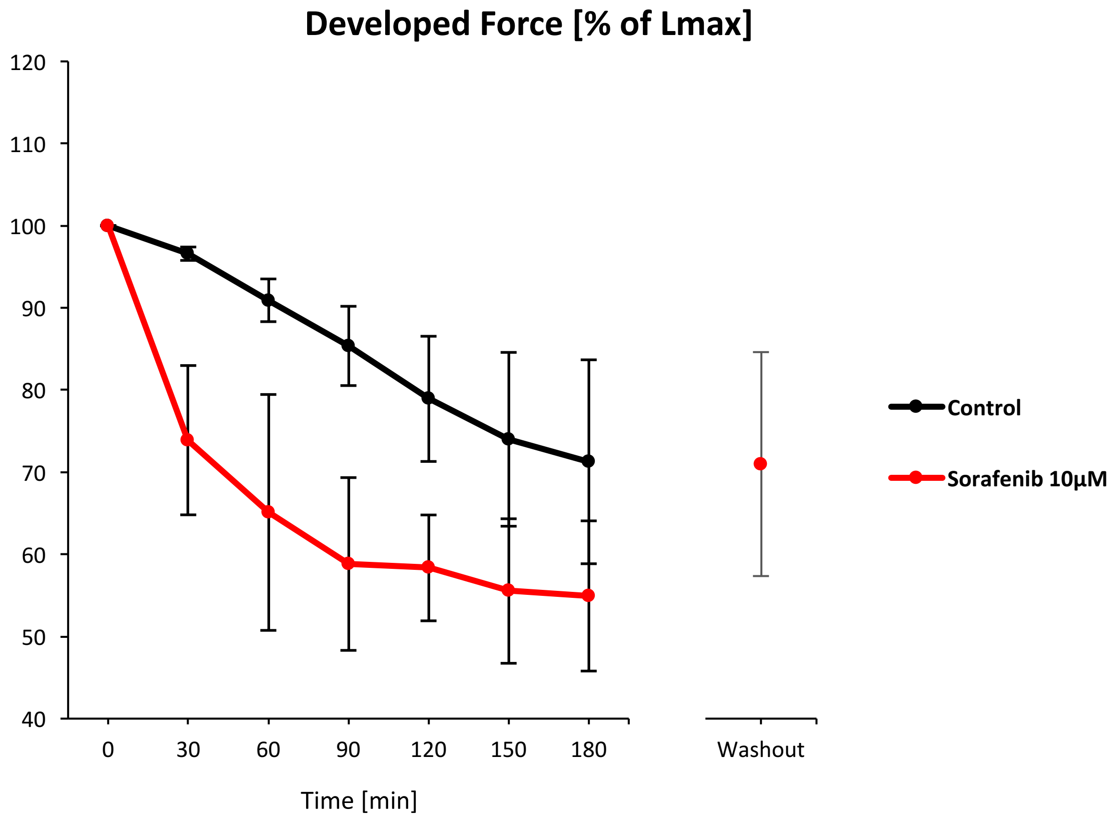

Fig. S1. Sorafenib-induced negative inotropy is sustained over several hours and remains reversible. n=3/4 trabeculae from 4 hearts, $p<0.05$ for all sorafenib time-points vs. baseline and for the 150- and 180-minute time points for control vs. baseline (inherent natural run-down of atrial muscle preparations in the organ bath). Developed force of sorafenib-treated muscles did not differ from control after wash-out ($p=0.94$).

Supplementary Figure S2

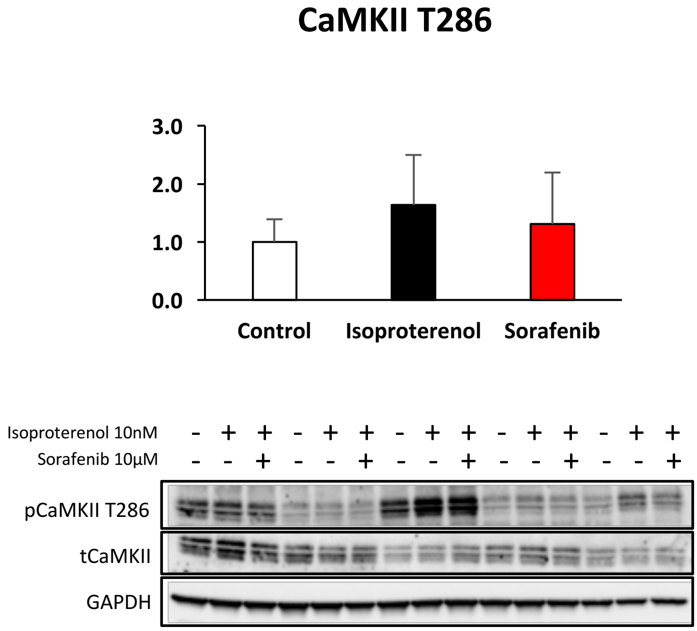

Fig. S2. CaMKII phosphorylation at threonine 286 is not acutely affected by sorafenib. Summary data (above) and original blots (below). n=5 hearts per group. For complete western blots see Supplementary Figure S4.

Supplementary Figure S3

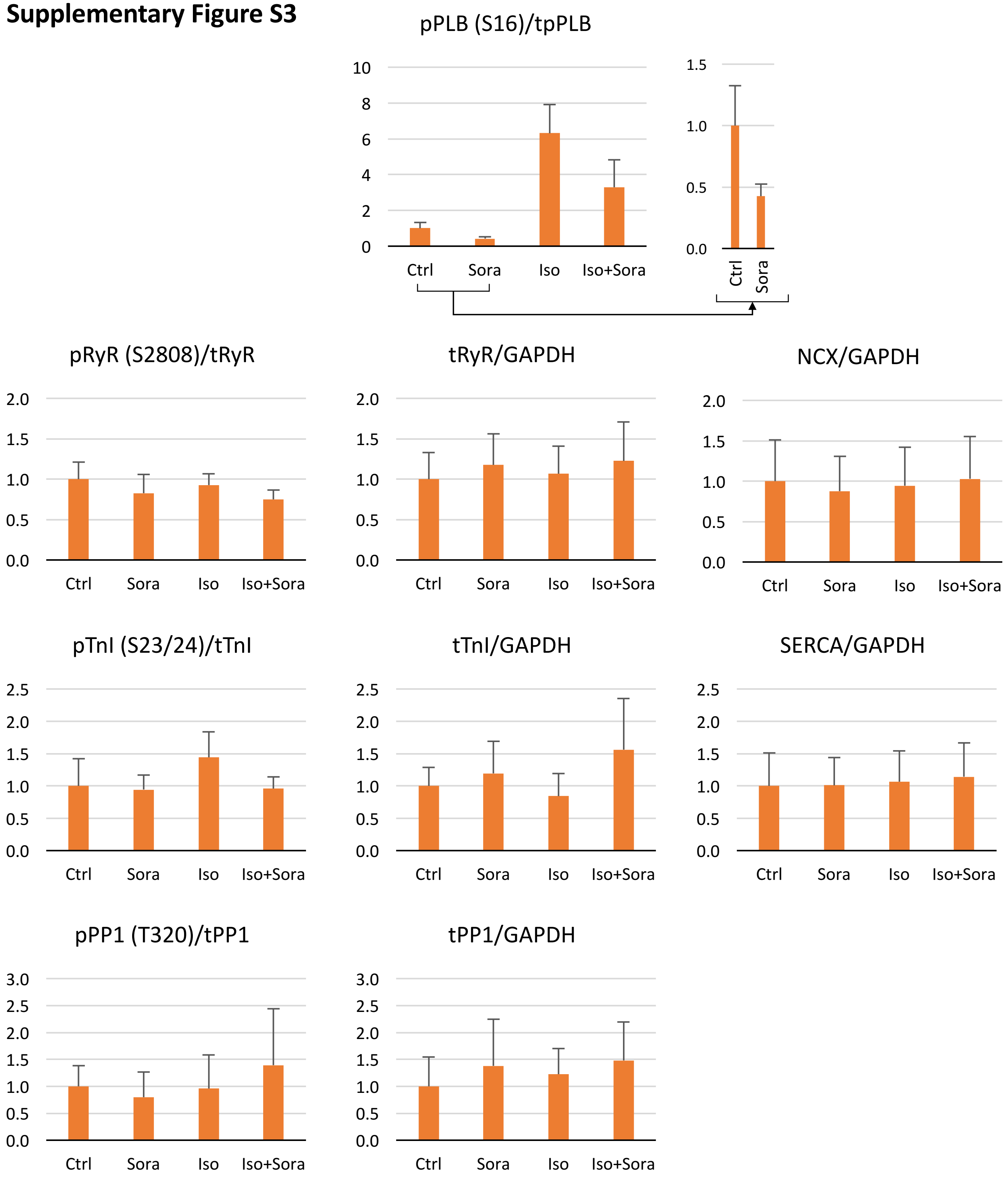

Fig. S3. Phosphorylation and expression of PLB, RyR, NCX, SERCA2a, PP1α, and troponin I in dependence of sorafenib (10µM) and/or isoproterenol (10nM). Adult murine cardiomyocytes were freshly isolated, pre-treated with sorafenib for 5 minutes and exposed to 10 minutes of isoproterenol; fold-changes are provided, n=4-6 hearts.

Supplementary Figure S4

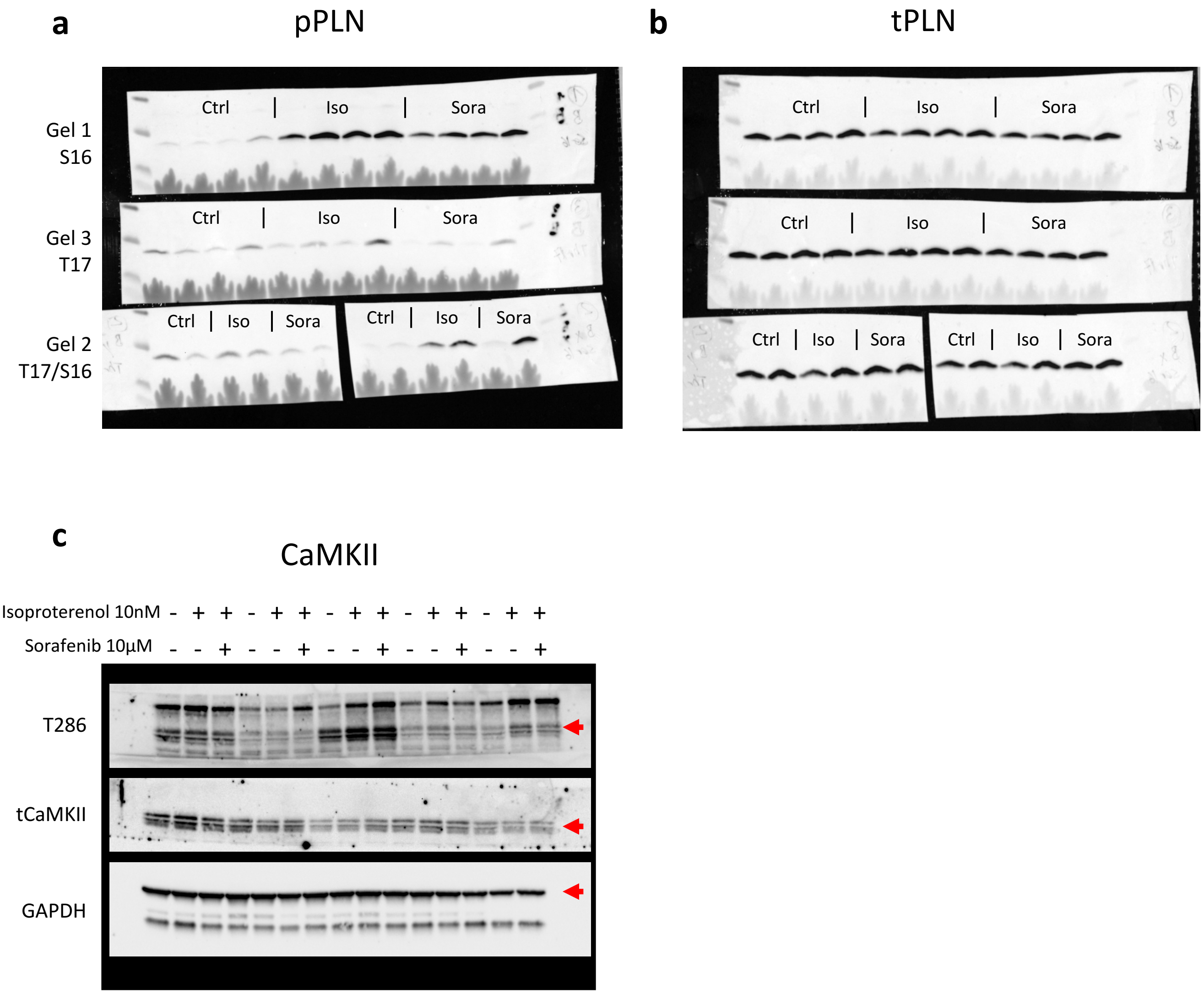

Fig. S4. Uncropped Western Blots. pPLN S16 and T17 (**a**), tPLN (**b**), pCaMKII T286, tCaMKII, and GAPDH (**c**).
